# Atlantic hurricane activity during the last millennium

**DOI:** 10.1038/srep12838

**Published:** 2015-08-05

**Authors:** Michael J. Burn, Suzanne E. Palmer

**Affiliations:** 1 Department of Geography and Geology, The University of the West Indies, Mona Campus, Kingston 7, Jamaica, W.I; 2 Centre for Marine Sciences, The University of the West Indies, Mona Campus, Kingston 7, Jamaica, W.I

## Abstract

Hurricanes are a persistent socio-economic hazard for countries situated in and around the Main Development Region (MDR) of Atlantic tropical cyclones. Climate-model simulations have attributed their interdecadal variability to changes in solar and volcanic activity, Saharan dust flux, anthropogenic greenhouse gas and aerosol emissions and heat transport within the global ocean conveyor belt. However, the attribution of hurricane activity to specific forcing factors is hampered by the short observational record of Atlantic storms. Here, we present the Extended Hurricane Activity (EHA) index, the first empirical reconstruction of Atlantic tropical cyclone activity for the last millennium, derived from a high-resolution lake sediment geochemical record from Jamaica. The EHA correlates significantly with decadal changes in tropical Atlantic sea surface temperatures (SSTs; r = 0.68; 1854–2008), the Accumulated Cyclone Energy index (ACE; r = 0.90; 1851–2010), and two annually-resolved coral-based SST reconstructions (1773–2008) from within the MDR. Our results corroborate evidence for the increasing trend of hurricane activity during the Industrial Era; however, we show that contemporary activity has not exceeded the range of natural climate variability exhibited during the last millennium.

The extent to which the climate dynamics of the Main Development Region (MDR) of Atlantic tropical cyclone activity are controlled by natural or anthropogenic climatic forcing factors remains unclear^1^,^2^. This uncertainty has arisen because of the reliance on historical meteorological records, which are too short to capture the natural long-term variability of climatic phenomena as well as a lack of understanding of the physical link between tropical Atlantic SSTs and tropical cyclone variability^3^,^4^. Consequently, the fifth assessment report of the Intergovernmental Panel on Climate Change (IPCC) concluded that there is low confidence in region-specific projections of tropical cyclone activity and that it remains uncertain whether recent changes in Atlantic tropical cyclone activity lie outside the range of natural variability^2^. While much progress has been made in climate-model projections of global tropical cyclone activity^1^, such uncertainty at the basin-wide scale makes it difficult for countries, particularly Small Island Developing States, to plan for the significant socio-economic consequences that are likely to arise due to 21^st^ Century global warming.

Atlantic tropical cyclones occur within the Atlantic warm pool, a region of warm water (>28.5 °C) that encompasses the Caribbean, the Gulf of Mexico, and the western tropical North Atlantic (Fig. 1) and varies spatially on interannual and interdecadal timescales^5^. Their activity is controlled by thermodynamic factors that govern heat exchange between the surface ocean and atmosphere, and dynamical factors that control vertical wind shear, a measure of the difference in average wind speed between the upper and lower layers of the atmosphere^5^,^6^,^7^,^8^. In general, a large (small) warm pool is associated with higher (lower) SSTs, tropospheric instability and precipitation, and lower (higher) vertical wind shear^5^ resulting in enhanced (subdued) tropical cyclone activity.

On longer timescales, Atlantic tropical cyclone activity is influenced primarily by the El Niño Southern Oscillation (ENSO) and the Atlantic Multidecadal Oscillation (AMO)^5^,^6^,^9^,^10^,^11^, which combine to modulate SSTs and vertical wind shear in the MDR. ENSO affects Atlantic climate dynamics on interannual (2–8yr) timescales and its amplitude has been shown to be modulated on interdecadal (50–90yr) timescales during the last millennium^12^. It influences the tropical Atlantic climate by controlling variations in sea level pressure across the equatorial Pacific, which modulate the strength of the Pacific Walker circulation^13^ and upper-level westerly wind flow into the Atlantic MDR^14^. The corresponding changes in vertical wind shear in the tropical Atlantic parallel changes in the strength of the Atlantic NE trade winds caused by fluctuations in the surface pressure gradient between the North Atlantic and equatorial Pacific. Thus in the MDR, El Niño (La Niña) events are typically associated with stronger (weaker) vertical wind shear, which suppresses (enhances) rainfall and tropical cyclone formation^11^,^15^. The AMO is a basin-wide index of Atlantic SST anomalies^16^, which varies periodically on interdecadal timescales (60–80 years) and controls the thermodynamic environment that underpins hurricane development^6^. Its influence on hurricane activity is manifested in the interdecadal behaviour of Atlantic Accumulated Cyclone Energy (ACE; 1851–2013)^5^,^6^, a composite measure of the frequency, intensity and duration of tropical cyclones^17^.

While contemporary tropical cyclone dynamics are relatively well understood, few observational records of their long-term natural variability extend back beyond the instrumental period. Existing historical documentary accounts rely heavily on colonial archives and are restricted to relatively narrow windows, spatially and temporally^18^,^19^,^20^,^21^. Similarly, of the few existing geological records of washover deposits within the MDR^22^,^23^,^24^,^25^, some have been shown to under-represent hurricane activity^26^ and their interpretation can be complicated by difficulties in distinguishing between storm and tsunami-induced events.

To avoid these shortcomings, we adopt a novel approach to the long-term reconstruction of Atlantic hurricane activity. We calibrate a high-resolution time series of lake level change from a previously published sediment geochemical record from Jamaica^27^ against the instrumental ACE index for the geographical area defined by the grid cell 15–25°N; 70–80°W for the period 1851–1969. While it has been established that the basin-wide ACE index underestimates tropical cyclone activity during the earlier parts of the record^28^, the passage of storms through the selected grid cell is well-sampled because the area encompasses Cuba, Jamaica, Hispaniola, the Bahamas, and the Cayman Islands, for which detailed meteorological and archival records are available back to the 19th Century (C. Landsea, pers. comm.). Thus, we argue the selected grid cell is both representative of activity in the region and relatively complete back to 1851. Given the established connection between precipitation and tropical cyclone activity in the MDR^29^ and the strong and statistically significant correlation between local rainfall, the sediment geochemical record and the ACE index on multiple timescales (see below), we demonstrate that the Jamaican time series is a robust proxy for hurricane activity and extend it back to the beginning of the last millennium. In doing so, we address the following research questions: (1) How does tropical cyclone activity change under contrasting mean climatic states of the northern hemisphere? (2) Are the interannual and interdecadal patterns of contemporary tropical cyclone activity persistent and stationary during the last millennium? (3) What does the extended ACE index reveal about the relative contributions of natural forcing factors to Atlantic tropical cyclone activity? (4) Do the observed trends in Atlantic tropical cyclone activity during the Industrial Era exceed their longer-term natural variability?

Our reconstruction of hurricane activity is derived from the third Principal Component (PC3) of mm-scale ITRAX μ-XRF core scans of a ~1200-year long sediment record of lake-level change from Grape Tree Pond in southern Jamaica^27^ (Figs 1,2a,3d and 4c). The pond is a freshwater-fed and anoxic mangrove lagoon, which is sensitive to precipitation variability on multiple timescales. High rates of sediment accumulation (~2.5 mm^−yr^) combined with low levels of bioturbation result in sedimentation rates sufficient to capture geochemical changes at an annual resolution. We improve the original ^14^C-based age model for the site by anchoring the PC3 record to the annually resolved coral-based SST reconstruction from the eastern Yucatan Peninsula^30^ for the period 1775–2009 (Fig. 1; see methods). Of the first three principal components of the geochemical data, PC1 explains 47.6% of the variance within the dataset and represents a gradient of sediment deposition from organic sediments to a combination of inorganic authigenic and detrital elements, the latter interpreted to reflect the aerial deposition of Saharan dust^27^. PC2 explains 11.9% of the variance and represents a salinity gradient. PC3 explains 8.9% of the variance and represents a gradient of redox conditions at the sediment-water interface of the lagoon and exhibits a positive relationship with lake level change^27^.

Historical rainfall measurements (1881–1966) from Easington, a town located 4.6 km to the northeast of Grape Tree Pond (Fig. 2a), demonstrate that more than 75% of average annual precipitation falls at the study site during the hurricane season (June-November; Fig. 2e) compared with 50% of average annual precipitation for Jamaica (Fig. 2b). Very little precipitation occurs outside of the hurricane season because of the influence of the rain shadow of the Blue Mountains. Comparison between rainfall measurements at Easington and the frequency of tropical cyclones passing through the grid cell 15–25°N; 70–80°W for the period 1893-1966, demonstrates a strong positive relationship (r = 0.64; p < 0.001; Fig. 2c) between the passage of storms and local rainfall. Further, qualitative comparisons between local rainfall and the basin wide ACE index show that the lowest total annual rainfall for Easington (561 mm) occurred in 1914, which coincided with the least active hurricane season on record as represented by a basin-wide ACE value of zero (Fig. 2d). In contrast, the record high for Easington (3148 mm) occurred in 1933, which coincided with the most active hurricane season during the 20^th^ Century (ACE ~ 259 10^4 ^kt^2^; Fig. 2c,f). Consequently, lake level change at Grape Tree Pond occurs in response to rainfall variability associated with the passage of storms during the hurricane season and we therefore interpret the PC3 series to reflect changes in tropical cyclone-induced precipitation.

The Jamaican PC3 series correlates significantly with different tropical cyclone-related environmental parameters across the MDR for the modern historical period on decadal and interdecadal timescales (Fig. 3; Table 1). Strong and statistically significant relationships exist between PC3 and the locally derived ACE index (15–25°N; 70–80°W) on decadal timescales (1851–1969; r = 0.63, r^2 ^= 0.4, p < 0.1) and basin-wide on high- (r = 0.71, r^2 ^= 0.5, p < 0.1) and low frequency (r = 0.9, r^2 ^= 0.81, p < 0.05) timescales from 1851–2010. It correlates significantly with two independent coral-based SST reconstructions from the Yucatan Peninsula^30^ and Venezuela^31^ (Fig. 3e,f; Table 1), which extend back to 1773 and 1918, respectively. Further qualitative similarities are observed between the low-frequency trends of the PC3 series and those of a SST reconstruction from Puerto Rico (Fig. 3g), which dates back to 1750^32^. Moreover, PC3 captures well-known historical climatological events including the interval of intense hurricane activity during the 1870 s and 1880 s and periods of quiescence during the 1850 s and 60 s and at the turn of the 20^th^ Century^33^,^34^. The apparent centennial-scale increase, and interdecadal variability of hurricane activity spanning the 20^th^ and early 21^st^ Centuries is supported by similar variability in Caribbean-wide SST (Fig. 3) and the PC3 time series. However, caution should be taken in the interpretation of trends exhibited by the basin wide ACE index, which becomes increasingly inaccurate prior to the advent of aircraft reconnaissance in 1944^28^.

Given the statistically significant relationships between hurricane activity (ACE), rainfall at Easington and the PC3 timeseries on different spatial and temporal scales, we calculate the Extended Hurricane Activity index (EHA) by regressing PC3 against the ACE index (15–25°N; 70–80°W) for the period 1851–1969 using a linear least squares model:


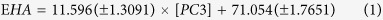


The EHA index (Fig. 4c) spans three distinct climatological intervals of the last millennium; the relatively warm mean climatic state of the Medieval Climate Anomaly (MCA; ~900–1350 CE), the relatively cool mean state of the Little Ice Age (LIA; ~1450–1850 CE), and the period of centennial warming, which distinguishes the Industrial Era since 1870. In general, average Caribbean-wide storm activity during the MCA was lower (EHA ~ 65.1 × 10^2 ^kt^2^) than that of the LIA (~73.3 × 10^2 ^kt^2^) and the 20^th^ Century average (EHA ~ 74.7 × 10^2 ^kt^2^; ACE ~ 67.3 × 10^2 ^kt^2^). However, hurricane activity during the LIA is characterised by a greater amplitude of variation where increased frequency of short intervals of enhanced and suppressed activity correspond to periods of low natural radiative forcing. The highest average levels of activity (EHA ~ 86 × 10^2 ^kt^2^; 1580–1650 CE) occurred during the late 16^th^ and early 17^th^ Centuries, a trend corroborated by a coupled ocean-atmosphere climate model simulation of annual basin-wide tropical cyclone counts^35^, which indicates heightened activity during the LIA (Fig. 4d). Further support for enhanced activity during the LIA comes from a record of hurricane deposits in a coastal karst basin in the Bahamas, which suggests heightened activity occurred between 1350 and 1650^25^. This important result suggests that average hurricane activity during the industrial period has not exceeded its longer-term natural variability during the last millennium. While the general increase in activity revealed by the EHA index is not consistent with the longer-term decreasing trend of hurricane counts simulated by the statistical model reconstruction driven by estimates of landfalling hurricane strikes from overwash sediment records (Fig 4d), the strong interdecadal variability exhibited by the EHA index is well represented in both^36^. This similarity is particularly striking during the modern historical period (1800 – present), where the records are characterised by decadal change superimposed on the longer term centennial increase in activity.

Comparison between the EHA index and indices of natural radiative forcing suggest changing solar irradiance has a significant influence on tropical cyclone activity. Spectral analysis of the previously published PC3 time series revealed periodicities consistent with those of changing solar irradiance (387, 208, 86, 23 years) combined with those of interdecadal variability (57 and 68 years)^27^ possibly associated with the AMO or the modulation of ENSO amplitudes on interdecadal timescales. A morlet wavelet analysis applied to PC3 (Fig. 4e) reveals that the interdecadal variability of hurricane activity (centred on a ~60-year periodicity) similarly exhibits strong and statistically significant power during intervals of low natural radiative forcing since the beginning of the LIA. Bandpass filters applied to PC3 to isolate interannual (5–8 year) and interdecadal (54–80 year) timescales, support this interpretation and suggest further that the variability is generally greater during radiative forcing minima on both high and low frequency timescales (Fig. 4f), a phenomenon that is particularly conspicuous during the so-called Spörer and Maunder grand solar minima. Taken together, our analyses suggest that interdecadal hurricane activity was subdued during the warm mean climatic state of the MCA. It subsequently became more pronounced during the LIA and, after a brief transitional state characterised by enhanced variability between 1850 and 1900, then returned to a more subdued state during the late industrial period (1950–2010), an episode of distinct climatic warming probably triggered by a combination of anthropogenic activity and a positive phase of the AMO^37^. Remarkably, the interdecadal behaviour of hurricane activity does not appear to persist as strongly during the warmer mean climatic states of the last millennium and therefore exhibits non-stationarity with respect to its response to changing mean climate states. While the changing magnitude of interannual variability is not significant at the 95% level it is nevertheless also clearly subdued during the early and latter parts of the record and more active during the LIA (Fig. 4f). The similarity in the response of interannual and interdecadal changes suggests that both ENSO and AMO may share a common response to deviations in external radiative forcing and raises the possibility that the variability of storms may become more subdued during the anthropogenically-forced warmer mean climate of the 21^st^ Century.

We propose that external forcing, whether solar, volcanic and/or anthropogenic, exerts a significant influence on Atlantic hurricane activity by its modulation of both ENSO and AMO. Thus our results support the hypothesis that changes in radiative forcing may influence ENSO dynamics by means of the ‘ocean dynamical thermostat hypothesis^38^,^39^,^40^’. Radiative cooling (warming) of the tropics reduces (increases) the zonal sea surface temperature gradient in the equatorial Pacific due to greater cooling (warming) of the western compared with the eastern equatorial Pacific^39^,^41^. The latter region is buffered from further cooling (warming) by strong coastal upwelling. The Walker circulation weakens (strengthens) as a consequence and an El Niño-like (La Niña-like) state develops in the tropical Pacific increasing (decreasing) upper-level westerly flow into the MDR of Atlantic tropical cyclones and in turn suppressing (promoting) rainfall and hurricane activity. Further observational support for the thermostat hypothesis comes from the Pacific-based Palmyra coral record^42^ which suggests not only that the Pacific SST gradient decreased during El Niño-like periods of the LIA, but also that enhanced ENSO activity dominated the mid-seventeenth century, consistent with our record of greater variability of hurricane activity in the tropical Atlantic. The trend to frequent and more intense El Niño-like conditions during the 17^th^ Century is further supported by lake-sediment evidence of drought conditions in the Yucatan peninsula of Mexico, the Dominican Republic and the Cariaco Basin, Venezuela^43^,^44^,^45^, during which subdued hurricane activity was likely.

Our results are also consistent with the external forcing of the AMO, which has been linked to tropical cyclone activity^5^,^46^,^47^ during the modern historical period. Recent climate model simulations^45^,^46^ link long-term AMO dynamics to changes in radiative forcing associated with the combined influence of solar and volcanic activity, possibly propagated through the Atlantic Meridional Overturning Circulation (AMOC) to influence SSTs and tropical cyclones in the North Atlantic. While further detection and attribution studies are required to establish the relative roles of external and internal forcing on tropical cyclone dynamics, the newly developed EHA index provides significant insights into the historical context within which 21^st^ Century hurricane activity may be understood.

## Methods

### Environmental data and filtering

**Figure 2:**
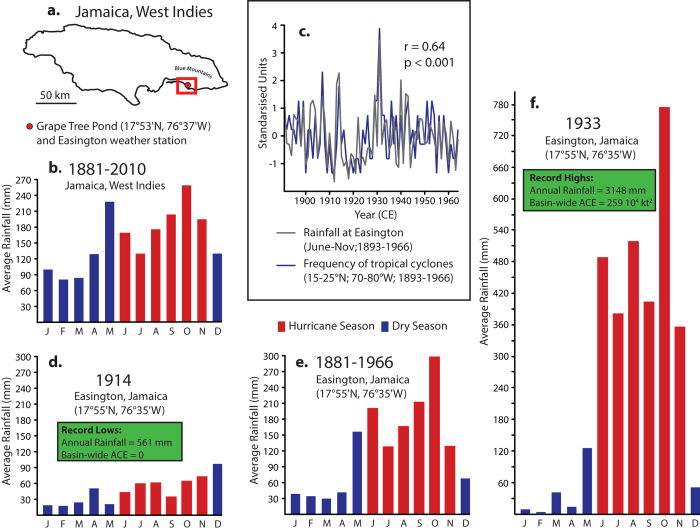
Precipitation data for Jamaica. (**a**) Map showing location of Grape Tree Pond in Jamaica and the location of Easington meteorological station in the Parish of St. Thomas. (**b**) Average rainfall (mm) for Jamaica (1881–2010). (**c**) Relationship between total summer rainfall (June-Nov) at Easington weather station and the frequency of known storms passing through the geographic area defined by the grid box 15–25°N, 70-80°W for the period 1893–1966. (**d**) Record low measurements of total rainfall at Easington and basin-wide hurricane activity during 1914. (**e**) Average rainfall measured at Easington for the period 1881–1966. (**f**) Record high measurements in total rainfall at Easington and basin-wide hurricane activity during 1933. Map created by Suzanne Palmer using Adobe Illustrator CC 2014.1.0 Release. Precipitation data provided by the Jamaican Meteorological Service.

**Figure 3:**
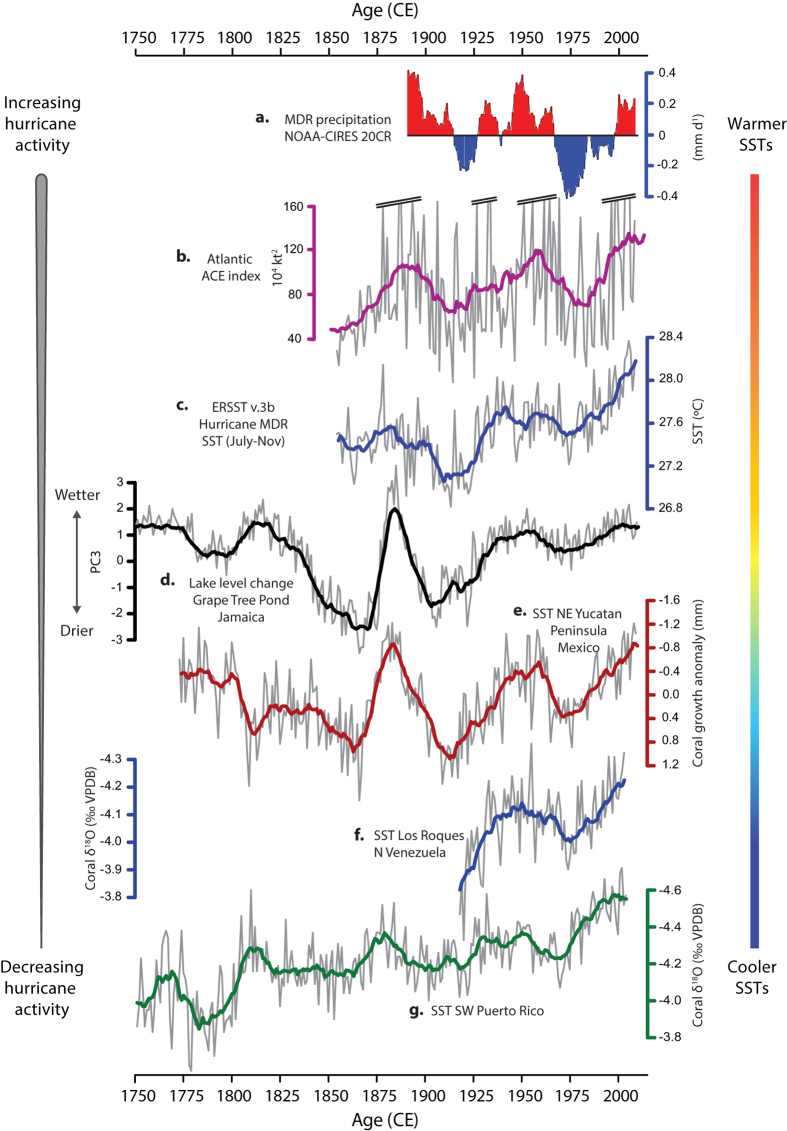
Comparison of environmental parameters and proxy-based reconstructions of MDR climatic phenomena during the modern historical period. (**a**) MDR precipitation for the interval 1871–2012 (modified from Dunstone^29^). Reprinted by permission from Macmillan Publishers Ltd: Nature Geoscience 6, 534-539, copyright 2013. (**b**) Revised Accumulated Cyclone Energy (ACE) index for the MDR from the National Hurricane Center’s Hurricane Database (HURDATv2). (**c**) MDR SSTs (July-November) from the NOAA NCDC extended reconstructed global sea surface temperature data (ERSSTv.3b)^49^. (**d**) PC3 sequence of lake-level change at Grape Tree Pond, Jamaica^27^. (**e**–**g**) Coral-based SST reconstructions from the Yucatan Peninsula, Mexico^30^, Los Roques, Venezuela^31^ and SW Puerto Rico^32^. All time-series were filtered using an 11-point running mean except (**b**) which was filtered using a 21-point running mean.

Average total annual precipitation data for Jamaica (1881-2010; Fig. 2) were provided by the Jamaican Meteorological Service and are based on an average of rain gauges collected from weather stations across the different Parishes of Jamaica. Some spatial bias is to be expected in these data particularly in the earlier part of the record (~1881–1900) when there were fewer weather stations. Average annual precipitation data for Easington, St. Thomas (1881–1966) were made available by NOAA Central library Data Imaging Project (CLDIP; http://docs.lib.noaa.gov/rescue/data_rescue_jamaica.html). Precipitation and SST data for the Main Development Region of hurricane activity were obtained from the NOAA Earth System Research Laboratory (ESRL) 2° × 2° Twentieth-Century Reanalysis (20CR; 1871–2008)^48^ and NOAA Extended Reconstructed SST version 3b ERSSTA v.3b; 1860–2008)^49^, respectively. Annual data were selected from the geographical area defined by the grid reference 10°–20°N, 15°–85°W and analyses were performed for the months June to November (Hurricane season). Data for the Accumulated Cyclone Energy (ACE) index, a composite measure of the frequency, intensity and duration of tropical cyclones (1851–2013) was obtained from the hurricane database re-analysis project (HURDATv2; http://www.aoml.noaa.gov/hrd/hurdat/Data_Storm.html). The Jamaican PC3 time series was obtained from the supplementary material accompanying the original study^27^ (http://onlinelibrary.wiley.com/doi/10.1002/jqs.2660/suppinfo). Data for the three coral-based SST reconstructions^30^,^31^,^32^ and those of solar and volcanic activity^50^,^51^,^52^ were recovered from the NOAA NCDC paleoclimatology database (http://www.ncdc.noaa.gov/data-access). All datasets represented in Fig. 3 were filtered using an 11-year running mean for the modern historical period (1750–2014) except for the ACE timeseries, which was filtered using a 21-year running mean.

### Chronology

**Figure 5:**
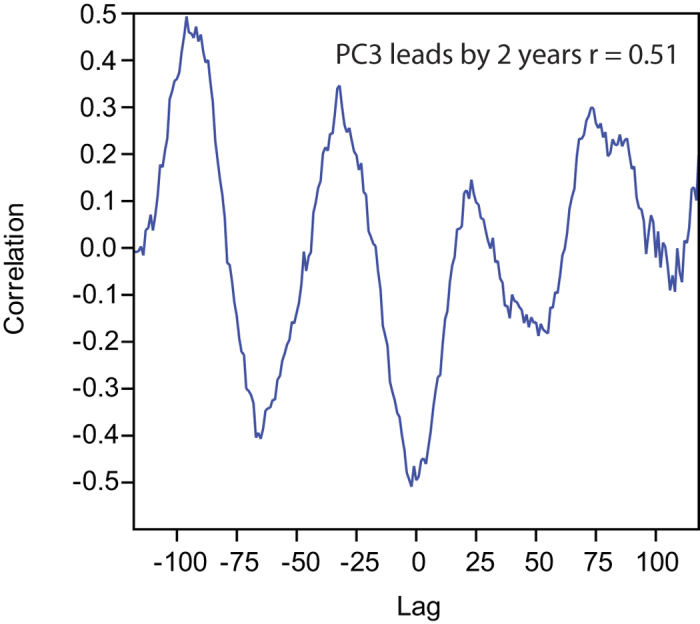
Cross-correlation between the annually-resolved coral growth rate record from the Yucatan Peninsula^30^ and Jamaican PC3 timeseries^27^ for the period 1773–2008.

The age model for the Jamaican PC3 climate series^25^ was harmonized to annual resolution to aid direct statistical comparison with other climate time series. Given the strong and significant correlation between the unfiltered and annually-resolved coral growth rate record from the Yucatan Peninsula^30^ and Jamaican PC3 timeseries^27^ (1773–2008; r = −0.6, p < 0.001; Table 1), the PC3 record was anchored to the coral chronology for the interval 1773–2008 providing excellent chronological control during the recent historical period. Harmonization of the two chronologies was performed objectively by calculating Pearson’s cross-correlation coefficients between the two time series and adjusting the PC3 age model by the calculated time lag of 2 years to achieve optimum correlation (r = −0.6, p < 0.001) between the series (Fig. 5). The small age differences between the original ^14^C-based age model and the harmonized model support the robustness of the original age model^28^ and lie well within its errors.

### Correlation and time series analyses

The Extended Hurricane Activity (EHA) index was calculated by a linear least-squares regression of the 11-year running means of the Jamaican PC3 timeseries^27^ and the ACE index calculated for the geographical area defined by 15–25°N, 70–80°W for the period 1851–1969 using equation (1). These spatial and temporal boundaries were selected for calibration in order to ensure our reconstruction is representative of hurricane activity around Jamaica and to capture the greatest range of values in both datasets. Correlation between environmental parameters was performed on annual datasets, which were smoothed using 5-, 11-, 21- and 41-yr running means. Since all of the data series exhibit serial autocorrelation and can be described by an autoregressive AR(1) process, the number of independent samples (degrees of freedom) are smaller than the sample size (***N***_*obs*_). Thus the number of effective degrees of freedom (*N*_*eff*_) was calculated according to ref. 53 using the equation:


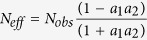


where *N*_*obs*_ is the number of observations and a_1_, a_2_ are the lag-1 autocorrelation coefficients for each time series. Uncertainty in the correlations was quantified by calculating *T* following ref. 54:


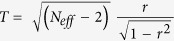


where r is the correlation and *N*_*eff*_ is the effective degrees of freedom. The distribution of T is assumed to have a t-distribution with N-2 degrees of freedom when the samples are not autocorrelated. This is used with a one-sided test to estimate the likelihood that the correlation has not occurred by chance.

After harmonization to annual resolution, the PC3 time series was detrended prior to analyses to suppress spectral leakage from low frequency (millennial) variations. A continuous wavelet transform was applied to the PC3 timeseries to detect significant periodicities and test for stationarity. The shape of the mother wavelet was set to morlet with a wave number ω_0 _= 6. Significance levels were calculated using a chi-squared test^55^ against a background of white noise. In order to capture inter-annual (ENSO) and interdecadal (AMO/ENSO) variability within the timeseries, the high and low frequency components of the PC3 time series were isolated using bandpass filters spanning interannual (5–8 year) and interdecadal (54–80 year) periodicities, All numerical analyses were performed using the software package PAST^56^.

## Additional Information

**How to cite this article**: Burn, M. J. and Palmer, S. E. Atlantic hurricane activity during the last millennium. *Sci. Rep.* **5**, 12838; doi: 10.1038/srep12838 (2015).

## Supplementary Material

Supplementary Dataset 1

## Figures and Tables

**Figure 1:**
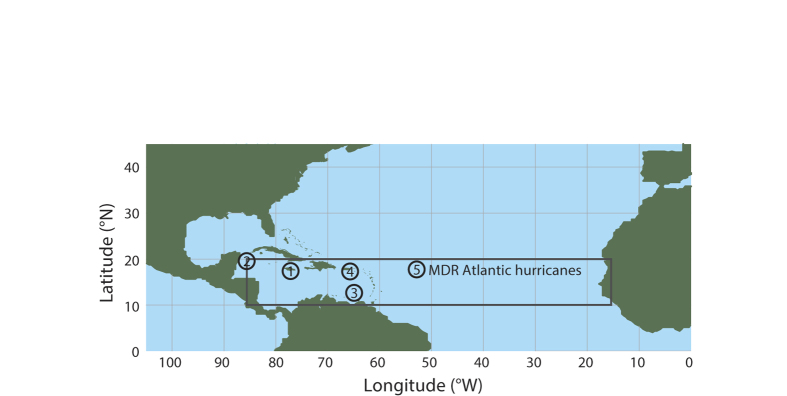
SST map of the tropical North Atlantic and Caribbean Regions. Map showing location of sites discussed in the text situated within the Main Developing Region (MDR; 10°–20°N; 15°–85°W) of Atlantic tropical cyclone activity. 1. Grape Tree Pond, Jamaica^27^; 2. Yucatan Peninsula, Mexico^30^; 3. Los Roques, Venezuela^31^; 4. SW Puerto Rico^32^ 5. MDR. Map created by Suzanne Palmer using Adobe Illustrator CC 2014.1.0 Release.

**Figure 4:**
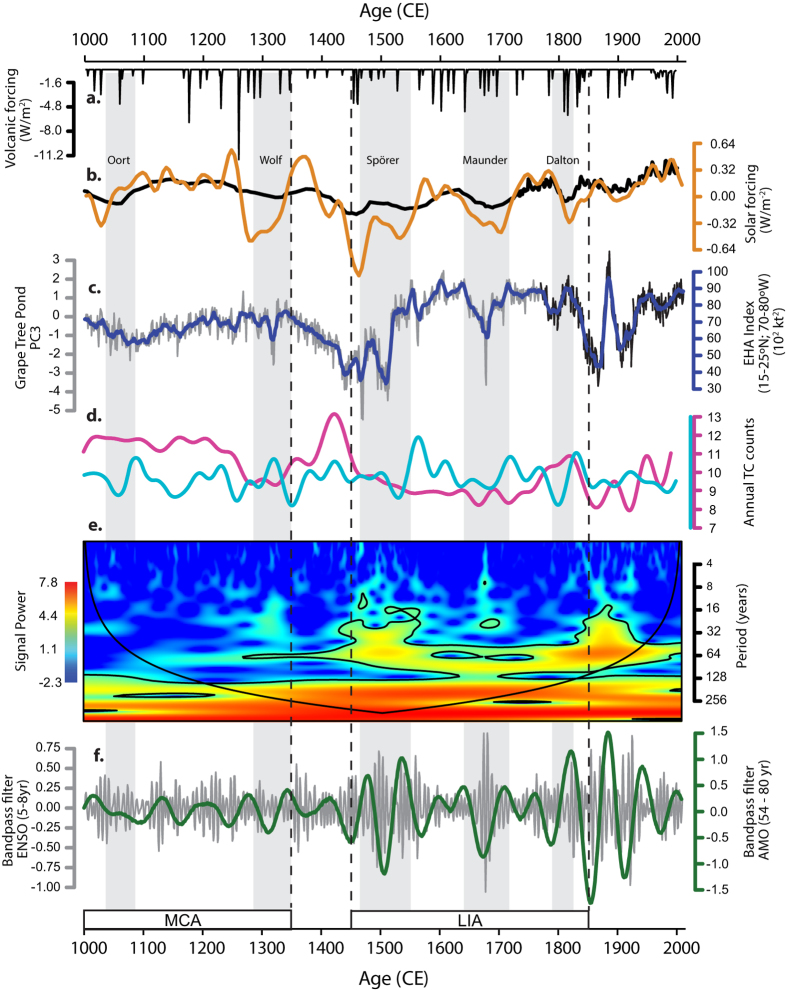
Time-series analyses of the Extended Hurricane Activity (EHA) index and comparison with natural external forcing (**a**) Changes in volcanic forcing^52^ (black line, upper panel) and (**b**) Total Solar Irradiance (TSI)^50^,^51^ (yellow and black). (**c**) PC3 sequence of lake-level change at Grape Tree Pond, Jamaica^27^ (grey) and the Extended Hurricane Activity (EHA) index (blue) calculated for the geographic region defined by the grid box 15–25°N, 70–80°W. The black line represents the section of the PC3 series anchored to the annually-resolved coral-based SST reconstruction from the Yucatan Peninsula^30^ (**d**) Annual basin-wide tropical cyclone counts from a coupled ocean-atmosphere climate model simulation^35^ (light blue), and from statistical model reconstructions driven by estimates of landfalling hurricane strikes from overwash sediment records^36^ (pink). (**e**) Morlet wavelet analysis applied to the PC3 sequence from Grape Tree Pond, Jamaica. Colours represent the signal strength, which is the squared correlation strength with the scaled morlet wavelet. The 95% confidence level is plotted as a contour and the cone of influence identifies the region of the wavelet spectrum in which boundary effects are present. (**f**) Bandpass filters applied to the PC3 sequence centred on interannual (5–8 years; grey) and interdecadal timescales (54–80 years; green). Intervals associated with the five grand solar minima are shaded in grey and those of the Medieval Climate Anomaly (MCA) and Little Ice Age (LIA) are represented in open boxes with dashed lines extended to the top of the figure.

**Table 1:** Correlation between the Jamaican PC3 series and the following environmental metrics of the Main Development Region (MDR) on inter-annual to decadal timescales (time-series were smoothed using 5-*, 11-^†^, 21-^‡^, and 41^ year running means, where indicated). Edf: Effective degrees of Freedom

Environmental parameters	Period (yrs)	Pearson’s r	r^2^	p-value	Edf
ACE index (15–25°N; 70–80°W)^†^	1851–1969	0.634	0.4	<0.1	5
Basinwide ACE index (10–20°N, 15–85°W)*	1851–2010	0.591	0.35	<0.05	10
Basinwide ACE index (10–20°N, 15–85°W)^†^	1851–2010	0.706	0.50	<0.1	5
Basinwide ACE index (10–20°N, 15–85°W)^‡^	1851–2010	0.816	0.67	<0.1	4
Basinwide ACE index (10–20°N, 15–85°W)^	1851–2010	0.902	0.81	<0.05	3
ERSSTAv.3b MDR (Jun–Nov; 10–20°N, 15–85°W)*	1854–2008	0.615	0.38	<0.1	5
ERSSTAv.3b MDR (Jun–Nov; 10–20°N, 15–85°W)^†^	1854–2008	0.677	0.46	<0.15	4
Coral-based SSTs (Los Roques, Venezuela)*	1918–2003	−0.836	0.7	<0.005	8
Coral-based SSTs (Los Roques, Venezuela)^†^	1918–2003	−0.956	0.91	<0.001	6
Coral growth-rate anomalies (Yucatan Peninsula)	1773–2008	−0.597	0.36	<0.001	26
Coral growth-rate anomalies (Yucatan Peninsula)*	1850–2008	−0.796	0.63	<0.1	4
Coral growth-rate anomalies (Yucatan Peninsula)^†^	1850–2008	−0.832	0.69	<0.15	3
